# Bioinformatics, Molecular Docking and Experiments *In Vitro* Analyze the Prognostic Value of CXC Chemokines in Breast Cancer

**DOI:** 10.3389/fonc.2021.665080

**Published:** 2021-05-26

**Authors:** Fei Wang, Chong Yuan, He-Zhen Wu, Bo Liu, Yan-Fang Yang

**Affiliations:** ^1^ Faculty of Pharmacy, Hubei University of Chinese Medicine, Wuhan, China; ^2^ Key Laboratory of Traditional Chinese Medicine Resources and Chemistry of Hubei Province, Hubei University of Chinese Medicine, Wuhan, China; ^3^ Collaborative Innovation Center of Traditional Chinese Medicine of New Products for Geriatrics of Hubei Province, Research Department of Material Basis of Traditional Chinese Medicine and Prescription, Wuhan, China; ^4^ Key Laboratory of Traditional Chinese Medicine Resource and Compound Preparation Ministry of Education, Hubei University of Chinese Medicine, Wuhan, China

**Keywords:** breast cancer, CXC chemokines, biomarkers, immune infiltration, molecular docking, quercetin, *in vitro*

## Abstract

The increasing incidence and mortality rate of Breast cancer (BC) make it a major public health problem around the world. CXC chemokines can mediate the migration of immune cells and regulate apoptosis in tumor. However, the expression and prognostic value of them in BC and their targeted drugs have not been clarified. Therefore, in this study, ONCOMINE, GEPIA2.0, UALCAN, Venny2.1.0, cBioPortal, STRING, Gene MANIA, Pathway Commons, DAVID6.8, Omicshare, Cytoscape3.6.1, TIMER2.0, Drug Bank, TCMSP, RSCBPDB, PubChem, pkCSM, Chem Draw, AutoDockTools-1.5.6 and PyMOL were utilized for analysis. The expression of CXCL1-3, CXCL9-13 between BC and normal tissues was significantly different in all the three databases. And the expression of CXCL1-2, CXCL12-13 was correlated with the stages of BC. But only CXCL1-3 were prone to mutation, and negatively correlated with survival and prognosis of BC patients. Taken together, CXCL1-2 might be therapeutic targets and biomarkers for BC patients. In addition, both of them were associated with immune infiltration. The results of molecular docking showed that Quercetin was most likely to be developed as drugs that interacted directly with CXCL1-2. And GLU29 of CXCL1, ASP-1, PRO-96, TRP-47 and LEU-45 of CXCL2 were the most potential sites, which provided valuable reference for further study of pharmacodynamics and mechanism. In addition, the inhibitory effect of Quercetin on proliferation and promoting apoptosis of BC related cell lines were confirmed *in vitro*. Western blot and Real-Time PCR confirmed that it increased the expression of CXCL1-2 in MDA-MB-231 and MCF-7 cells.

## Introduction

Breast cancer (BC) is a kind of malignant tumor caused by genes’ mutation of mammary gland epithelial cells with a variety of carcinogenic factors, resulting in cell proliferation out of control, and its mortality rate is in the forefront of all kinds of tumors (1). The incidence number of BC is about 2 million 880 thousand per year and the incidence rate of BC in China ranks 120 around the world (2, 3). BC has the characteristics of high degree of malignancy, rapid proliferation, easy metastasis and recurrence. At present, chemotherapy, surgery and medication are the most commonly used treatments for BC. However, due to the rapid progress of it, many patients lost the opportunity of early surgery (4). At the same time, some patients are prone to tolerance to conventional chemotherapy drugs, resulting in poor treatment effect (5, 6). In recent years, many studies have shown that the occurrence and development of BC are accompanied by the disorder of many genes, but the mechanism is still unclear (7, 8). Therefore, it is of great significance to explore therapeutic targets and prognostic markers for BC patients, and to elaborate the related molecular mechanisms for improving the effect of diagnosis and treatment.

Chemokines are named for their small molecular weight, four conserved cysteine residues and directional chemotaxis (9). Up to now, 48 chemokines and 18 signal transduction receptors have been identified in human. CXC chemokines are important members of this family (10). Tumor cells, immune cells and stromal cells in tumor microenvironment can secrete CXC chemokines and regulate their expression patterns, thus affecting the angiogenesis, occurrence, progression and metastasis of tumor (11). However, the expression and prognostic value of them in BC and their targeted drugs have not been clarified.

**Figure 1:**
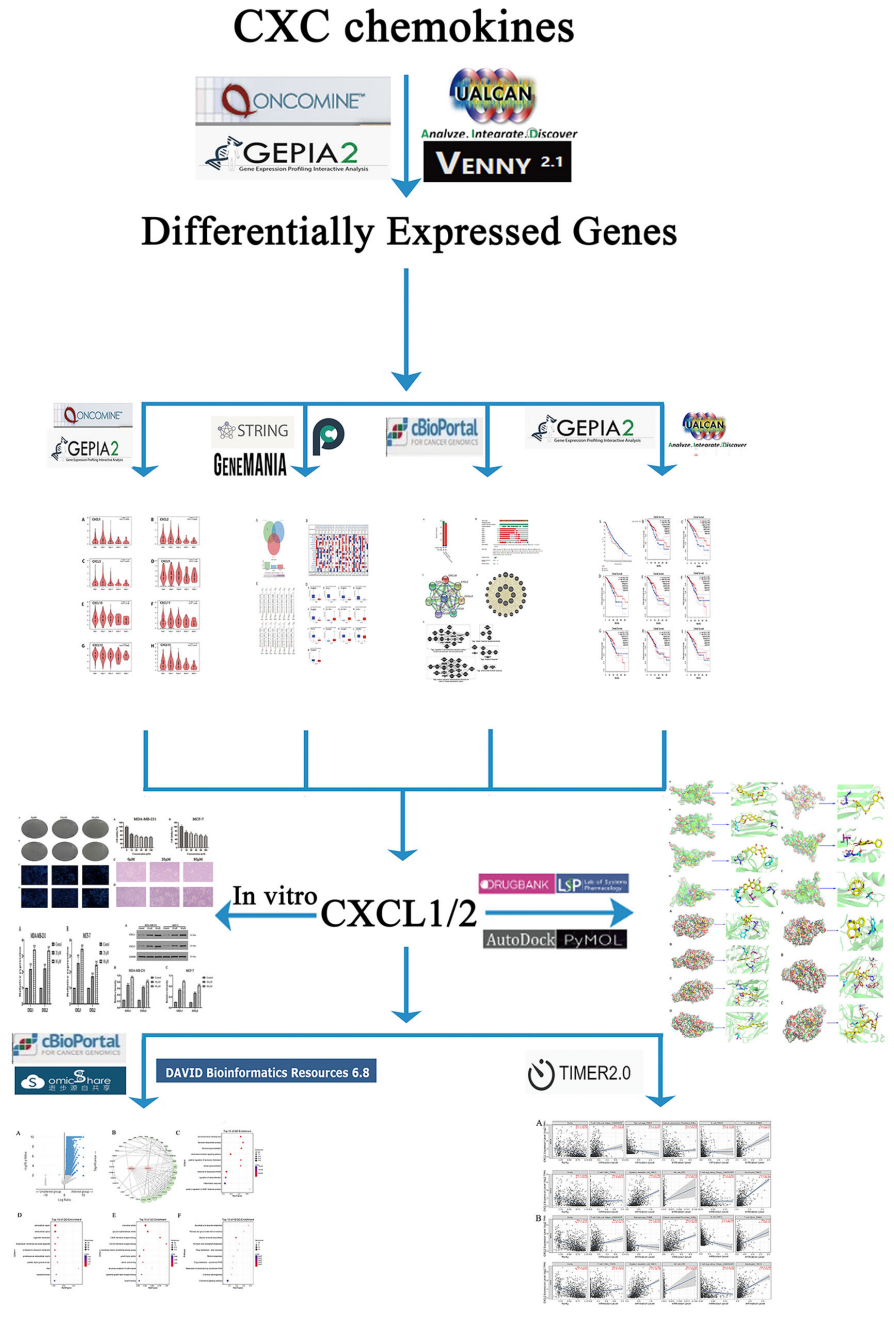
The flow chart of this study.

Therefore, we conducted a comprehensive bioinformatics analysis of CXC chemokines’ expression, mutation and their relationship with prognosis of BC patients through several large public databases. The biomarkers were selected according to the relationship between their expression and the survival of BC patients. Then, a network of biomarkers and their adjacent genes was constructed to analyze their biological functions and pathways. The relationship between biomarkers and immune cells was analyzed, at the same time. Finally, molecular docking and ADEMT were used to screen the targeted drugs of CXCL1-2. And experiments *in vitro* were used to confirm the above results. This study provided two new biomarkers and corresponding drugs for BC. The flow chart of this study is shown in **Figure 1**.

## Materials and Methods

### ONCOMINE Database Analysis

ONCOMINE (http://www.oncomine.org ) is a database built by the University of Michigan for drugs’ development and clinical practice. It has 12764 normal and 86733 tumor samples, which used to analyze the expression of target genes in different cancers (12). Through “differential analysis” module, students’ t-test was used to analyze the differential expression of CXC chemokines in BC and normal tissues by setting the significance threshold of *P* < 0.05, multiple change of 2 and gene ranking in the top 10%. The samples involved 10 studies (13–22).

### GEPIA2 Database Analysis

GEPIA2 (http://gepia.cancer-pku.cn/index.html) is developed by Peking University. It contains sequence expression data of 9736 tumors and 8587 normal tissues from TCGA and GTEX projects (23). In this study, “Single gene analysis” module was used to analyze the differential expression of CXC chemokines in BC and normal tissues based on TCGA normal and GTEx data. And their expression of different stages in BC was analyzed by setting |log2fc| cutoff to 1 and Q-value cutoff to 0.05 with ANOVA method. The overall survival (OS) rates of CXC chemokines were analyzed by “survival analysis” in GEPIA2, and the hazards ratio based on Cox pH model was calculated. The 95% CI were added as dotted line.

### UALCAN Database Analysis

UALCAN (http://ualcan.path.uab.edu/analysis.html) is a comprehensive database that provides analysis of comics data (including TCGA and MET500 queue data) which based on high-quality graphics using JavaScript and CSS (24). So, the “TCGA gene analysis” module of UALCAN was used to analyze the data onto the expression of CXC chemokines in BC and normal tissues. The different tumor stages and the survival rate of patients were analyzed at the same time. Students’ t-test was used to set the significance threshold of *P* < 0.05.

### cBioPortal Database Analysis

cBioPortal (http://www.cbiobortal.org) is a comprehensive database which developed by MSK to analyze multidimensional cancer genome data (25). Based on TCGA, 9536 samples of 18 BC studies are analyzed. Mutation data of CXC chemokines were obtained from “Mutation” through cBioPortal. The overall survival rates of differentially expressed genes and the data of adjacent genes co-expressed with them were obtained by “Comparison/Survival”. The Z-score of mRNA expression (RNA SEQ v2rsem) and protein expression Z-score (RPPA) were obtained with ± 2.0 as Z-score threshold.

### Neighbor Genes’ Networks, Pathways and Interactions of Differentially Expressed Genes

STRING (https://string-db.org/) is a database developed by European Molecular Biology Laboratory on functional association for genes (26). It includes 5090 species, more than 20 million proteins and 3 billion interactions. Gene MANIA (https://genemania.org/) is a real-time multi association Network Ensemble Algorithm for predicting functional association data related to input genes (27). Pathway Commons (http://www.pathwaycommons.org/) contains data from 9 databases, with more than 1400 pathways and 687000 interactions (28). The data onto neighbor genes and pathways related to differentially expressed genes were collected and integrated *via* them.

### Timer Database Analysis

TIMER (https://cistrome.shinyapps.io/timer/) is a tool for systematic analysis of immune infiltration in more than 30 cancer types (23). “Immune Association” was used to assess the correlation between biomarkers and immune cells in this study.

### Gene Ontology and KEGG Pathway Enrichment Analysis

Gene Ontology (GO) is a standardized classification system of genes’ function which used to comprehensively describe the properties of genes and their products in organisms. Kyoto Encyclopedia of Genes and Genomes (KEGG) is a system used to comprehensively analyze the information, function and relationship with targets of pathways. DAVID6.8 (https://david.ncifcrf.gov/summary.jsp) provides a comprehensive set of functional annotation tools for investigators to analyze the GO enrichment and KEGG pathway annotation involved in the targets (29).

The genes related to biomarkers obtained from cBioPortal were inputted into DAVID6.8, and species were selected as “Homo species” for GO enrichment and KEGG pathway analysis. Significant data were screened according to *P <* 0.05.

### Screening Drugs for Biomarkers

Both of the TCMSP (https://tcmspw.com/tcmsp.php) and Drug Bank (https://go.drugbank.com/) are databases containing information on a variety of natural compounds and corresponding genes (30, 31). RSCBPDB (https://www.rcsb.org/) is a database contains thousands of genes (32). pkCSM (http://biosig.unimelb.edu.au/pkcsm/prediction) is a database used to analyze the ADMET parameters of compounds, so as to discovery new drugs (33). Autodocktools-1.5.6 is a semi flexible molecular docking software and PyMOL is a software used to process the structure of small molecules and proteins before and after docking. It is generally believed that the binding strength of a compound to protein is inversely proportional to the binding energy, and its threshold is -4 kcal/mol. Meanwhile, according to ADMET principle, the possibility of the above drugs was analyzed.

### Cell Culture

MDA-MB-231 and MCF-7 cells were obtained from Hubei University of Chinese Medicine (Wuhan, China). The cells were cultured in DMEM (Gibco, MD, USA) supplemented with 15% fetal bovine serum (FBS) (Gibco, MD, USA) and 100 U of penicillin G with 100 µg of streptomycin per ml. Cells were incubated at 37°C under 5% CO2 in a humidified atmosphere.

### Cell Proliferation Assay

MDA-MB-231 and MCF-7 cells were incubated in 96-well plates for 24 h at a density of 5.0 × 10^3^ cells per well with five holes. And cells were treated with different concentrations (0, 10, 20, 40, 80 and 160 µM) of Quercetin for 24 h. Then, 20 µl MTT (Sigma, MO, USA) was added to each well for another 4 h. After the supernatant was removed, the formazan product obtained was dissolved in 150 µl dimethyl sulfoxide (DMSO) (Sigma) on a QB-9001 Microporous Quick Shaker (Kylin-Bell Lab Instruments Co. Ltd., Jiangsu, China) for 15 min. The absorbance was read at 490 nm with a Spark 10M microplate reader (Tecan, Männedorf, Switzerland).

### Plate Cloning Experiment

MDA-MB-231 and MCF-7 cells in logarithmic growth phase were inoculated into 6-well plates at 500 cells/well. They were divided into three groups and added 0, 20 and 80 µM Quercetin, respectively. 24 h after administration, fresh culture medium was added, and the culture medium was changed regularly within 14 days. On day 15, they were fixed with 4% polyoxymethylene for 10 min, washed 3 times with PBS, stained with Giemsa stain solution for 15 min. Then, the microscopes were cleaned with flowing water for observation and photographing.

### Hoechst 33342 Staining

MDA-MB-231 and MCF-7 cells were incubated with Quercetin (0, 20 and 80µM) for 24 h. Then, the cells were fixed with 4% polyoxymethylene for 15 min, washed 3 times with PBS, incubated with 1mL/well Hoechst 33342 (Beyotime, 8 µg/ml) for 15 min and then washed 3 times with PBS. Cells were observed with a fluorescence microscope.

### Western Blot Analysis

MDA-MB-231 and MCF-7 cells were seeded in 6-well plates at 5.0 × 10^5^ cells/well and treated with Quercetin (0, 20 and 80µM) for 24h. Then, cells in each well were photographed under 10x microscope. And the cell lysis was performed using RIPA lysis kit (AS1004, ASPEN, Wuhan, CHINA) and mixed with phenylmethanesulfonyl fluoride. Total protein quantification of the whole cell lysate was performed using BCA protein assay kit (AS1086, ASPEN, Wuhan, CHINA). The same total proteins were separated on 15% SDS-PAGE gel electrophoresis (AS1012, ASPEN, CHINA) and subsequently electrophoretically transferred onto a nitrocellulose membrane by electrophoresis (DTT-6C, DYCZ-400D, DYCZ-24DN, Beijing, CHINA). Membranes were blocked with 5% BSA for 2 h at room temperature, then incubated overnight at 4°C with primary antibodies, including CXCL1 (1:100 dilution, AB206411, ABCAM, UK) and CXCL2 (1:500 dilution, BS-1162R, BIOSS, Wuhan, CHINA). Membranes were washed 3 times, then incubated with HRP-Goat anti Rabbit (AS1107, ASPEN, Wuhan, CHINA). 1 h later, membranes were washed 3 times with tris buffered saline + Tween (TBST) for 10 minutes. Proteins were visualized by the addition of ECL (AS1059, ASPEN, Wuhan, CHINA), and membranes were scanned and imaged by the Fluor Chem FC3 system (Protein Simple, USA).

### Real-Time PCR Analysis

According to the manufacturer’s guidelines, total RNA was extracted from each group of cells by TRI pure reagent (EP013, ELK Biotechnology, Wuhan, CHINA). The primers of genes were designed and synthesized by PINUOFEI Biotechnology Co., Ltd (Wuhan, CHINA). Then, EnTurbo™ SYBR Green PCR Super Mix (ELK Biotechnology, Wuhan, CHINA) and Step One™ Real-Time PCR (Life Technologies, USA) were used to determine the mRNA expression of genes. The following thermal circulator conditions were used: pre-denaturation at 95°C for 3 min, denaturation at 5°C for 10 s, and denaturation at 58°C for 30 s, and denaturation at 72°C for 30 s (denaturation for 40 cycles). The melting curve condition was the default setting of the instrument. GAPDH was used as internal reference to normalize the expression of gene. Relative quantification was determined by 2-ΔΔCT method.

### Statistical Analysis

In this study, ANOVA was used to analyze the expression of CXC chemokines in GEPIA2.0 database and their relationship with the stages of BC. |Log2fc | cutoff < 1 and Q-value < 0.05 were considered to be significant. Overall survival (OS) was analyzed based on the Mantel–Cox test and Log rank *P* < 0.05 were considered to be significant. The data in ONCOMINE and UALCAN were analyzed by students’ t-test, and *P* < 0.05 was the significance threshold. Date in cBioPortal database were obtained with ± 2.0 as Z-score threshold. The partial Spearman’s correlation was used in TIMER database analysis. When |Rho| > 0.1, it indicated that there was a correlation between the genes and immune cells. Significant data in GO and KEGG pathway enrichment were screened according to *P* < 0.05 with students’ t-test. Relevant data of *in vitro* experiment were performed using SPSS 22.0 software (IBM, USA). Data were expressed as the mean ± SD. All experiments were repeated in triplicate and *P* < 0.05 was considered significant.

## Result

### Expression of CXC Chemokines in BC and Normal Tissues

**Figure 2:**
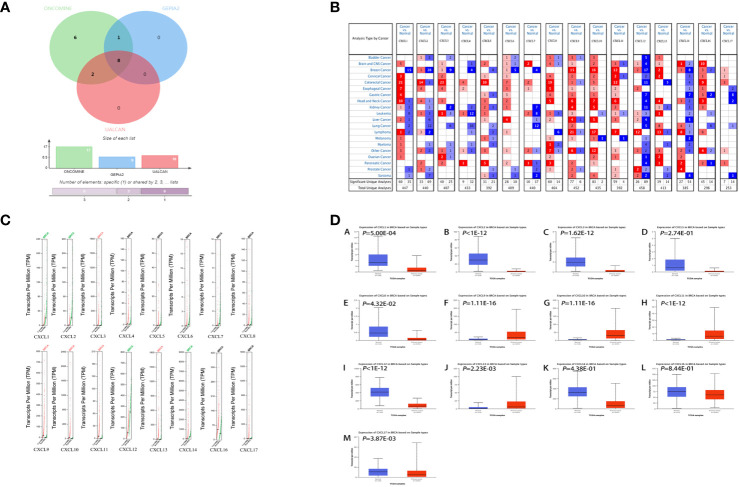
Differentially expressed CXC chemokines. **(A)** Wayne diagram of three databases’ results. **(B)** Differentially expressed CXC chemokines from ONCOMINE database. **(C)** Differentially expressed CXC chemokines from GEPIA2.0 database. **(D)** Differentially expressed CXC chemokines from UALCAN database. Samples were shown in (**B–D**) ANOVA was used to analyze the expression of CXC chemokines in GEPIA2.0 database and their relationship with the stages of BC. |Log2fccutoff < 1 and Q-value < 0.05 were considered to be significant. The data in ONCOMINE and UALCAN were analyzed by students’ t-test, and *P* < 0.05 was the significance threshold.

In order to screen CXC chemokines with significant difference between BC and normal tissues, 16 genes (excluding CXCL15) were analyzed based on ONCOMINE (54 BC and 38 normal samples), GEPIA2.0 (1085 BC and 291 normal samples) and UALCAN (1097 BC and 114 normal samples) databases, respectively. The results of these three databases were slightly different from each other. In ONCOMINE, there were 5 up-regulated genes and 9 down-regulated genes in BC (**Figure 2B**). In GEPIA2.0, there were 4 up-regulated genes and 5 down-regulated genes in BC (**Figure 2C**). In UALCAN, there were 4 up-regulated genes and 6 down-regulated genes in BC (**Figure 2D**). The intersecting genes of the three databases were obtained by Venny2.1.0 database, CXCL1-3 and CXCL12 were down-regulated in BC, and CXCL9-11, CXCL13 were up-regulated in BC (**Figure 2A**).

### Relationship Between Differentially Expressed Genes and Their Pathways

**Figure 3:**
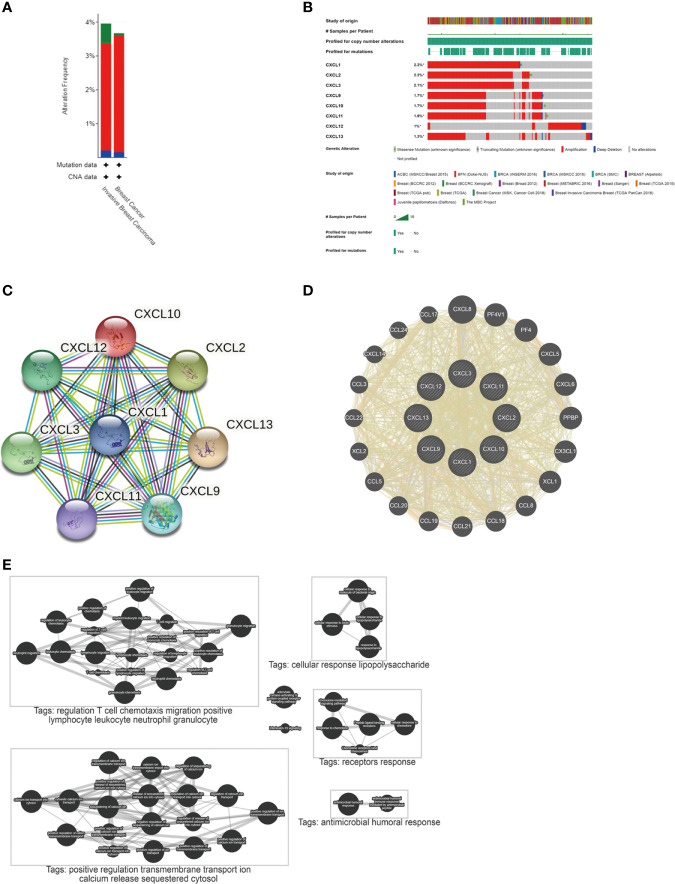
Mutation and interaction of differentially expressed CXC chemokines. **(A)** The mutation of genes in BC and invasive BC from cBioPortal. **(B)** Mutation of differentially expressed genes from cBioPortal. **(C)** A PPI network from STRING. **(D)** Circular diagram of differentially expressed genes from Gene MANIA. **(E)** Pathways involved in differentially expressed genes from Pathway Commons.

The interaction between differentially expressed CXC chemokines and their co-expressed genes was explored. A Protein-Protein Interaction (PPI) network **(** **Figure 3C** **)** and circular graph (**Figure 3D**) were obtained *via* STRING and Gene MANIA, respectively. As expected, 8 nodes and 138 edges were obtained in PPI network. Through plug-in analysis, CXCL1-2 and CXCL10-11 were found to have higher degree than others. In the circular graph, 28 nodes were obtained, 20 of which were closely related to differentially expressed genes. The functions of these genes were mainly related to receptors response, cellular response lipopolysaccharide, regulation T cell chemotaxis migration positive lymphocyte leucocyte neutral granulocyte, etc. (**Figure 3E**). And this result was consistent with our understanding.

### Mutation of Differentially Expressed Genes and Their Relationship With Stages In BC

The cBioPortal database was used to investigate the mutation of differentially expressed genes in 9115 BC patients from 18 studies. As a result, CXCL1-3, CXCL9-13 were altered in 2.2, 2.2, 2.1, 1.7, 1.7, 1.8, 1.0 and 1.3% of the queried BC cases. At the same time, through the cancer types summary, the minimum total cases as 1000 and the minimum altered cases as 2% were set to analyze the mutation of genes in BC and Invasive BC. Summary for BC, genes altered in 3.67% of 5204 BC cases, including mutation 0.08% (4 cases), amplification 3.44% (179 cases) and deep deletion 0.15% (8 cases). Summary for Invasive BC, genes altered in 3.96% of 1920 cases, including mutation 0.57% (11 cases), amplification 3.18% (61 cases) and deep deletion 0.21% (4 cases). It showed that the high mutation of invasive BC might be the main cause of the increased mortality. See **Figures 3A, B** for details.

**Figure 4:**
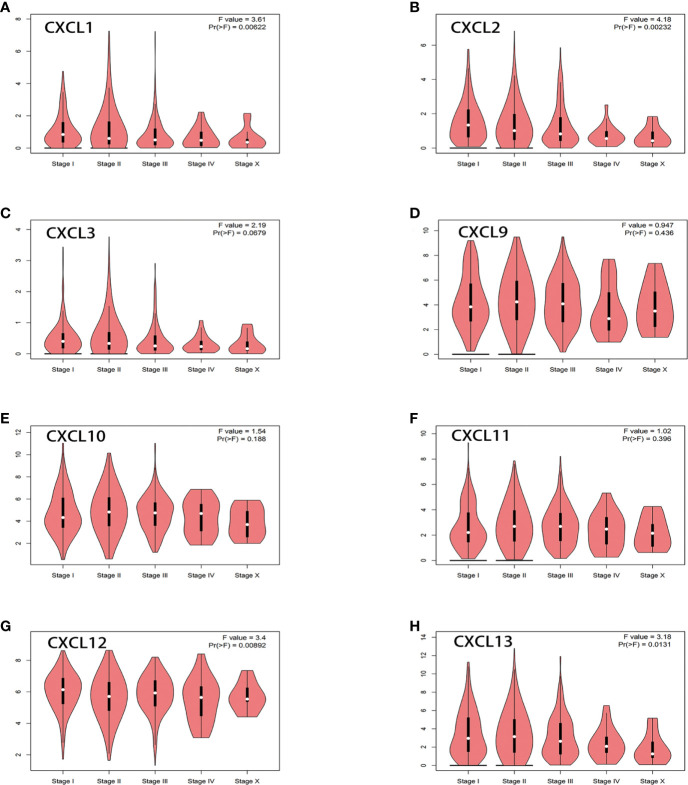
Relationship between differentially expressed genes and the stages in BC from GEPIA2.0. ANOVA was used to analyze multiple datasets, and Pr (>F) = 0.05 was the significance threshold. **(A)** CXCL1. **(B)** CXCL2. **(C)** CXCL3. **(D)** CXCL9. **(E)** CXCL10. **(F)** CXCL11. **(G)** CXCL12. **(H)** CXCL13.

Then, GEPIA2.0 and UALCAN were used to evaluate the relationship between differentially expressed genes and the stages in BC. As shown in **Figure 4**, there were no significant correlation between CXCL3 (PR (> F) = 0.0679), CXCL9 (PR (> F) = 0.436), CXCL10 (PR (> F) = 0.188), CXCL11 (PR (> F) = 0.396) and pathological stages of BC. However, CXCL1 (PR (> F) = 0.00622), CXCL2 (PR (> F) = 0.00232), CXCL12 (PR (> F) = 0.00892), CXCL13 (PR (> F) = 0.0131) were significantly correlated with the pathological stages of BC. These data indicated that CXCL1-2, CXCL12-13 were easily mutated in BC and they were closely related to the occurrence and development of BC.

### Relationship Between Differentially Expressed Genes and Survival of BC Patients

**Figure 5:**
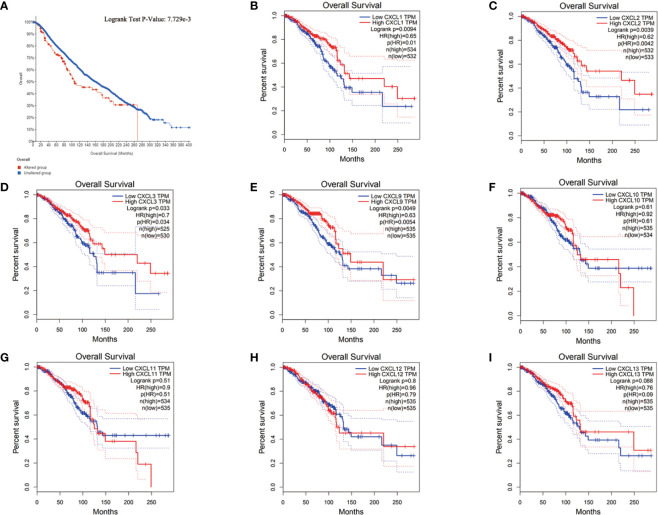
The relationship between differentially expressed genes and prognosis of BC. **(A)** The relationship between all differentially expressed genes and the prognosis of BC from cBioPortal. **(B–I)** The relationship between each differentially expressed gene and the prognosis of BC from UALCAN. Samples were shown in figures, and all of them used students’ t-test, *P* < 0.05 was the significance threshold.

In order to understand the relationship between differentially expressed genes and prognosis of BC patients, GEPIA2.0 (1070 BC patients), UALCAN (1081 BC patients) and cBioPortal (9115 BC patients) were used for comprehensive analysis. As shown in **Figure 5A**, the overall increase in differentially expressed genes was significantly associated with poor overall survival (P = 7.7729e-3). In addition, the down-regulation of CXCL1 (logrank P = 0.0094), CXCL2 (logrank P = 0.0039) and CXCL3 (logrank P = 0.033), and the up-regulation of CXCL9 (logrank P = 0.0049) were significantly associated with poor overall survival in BC patients (**Figures 5B–I**).

Based on the previous results, CXCL1-2 played an important role in all kinds of analysis. Therefore, they could be used as new biomarkers of BC.

### Immune Cell Infiltration of CXCL1-2 in BC

The occurrence and development of tumors were closely related to immunity. Furthermore, the correlation between the expression of CXCL1-2 with immune infiltration in BC from TIMER2.0 was investigated.

**Figure 6:**
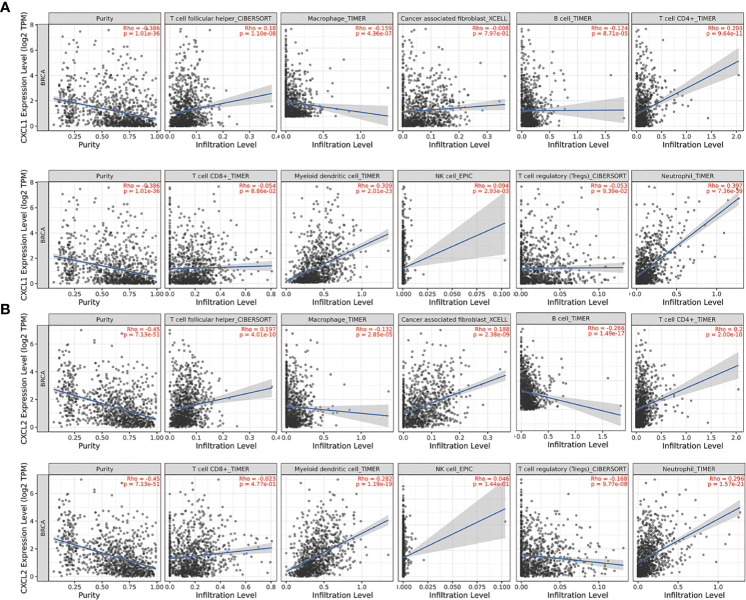
The relationship between the expression of two markers and the expression of immune invasion in BC from TIMER2.0. The partial Spearman’s correlation was used in TIMER database analysis, n=1100. When |Rho| > 0.1 and *P* < 0.05, it indicated that there was a correlation between the genes and immune cells. In general, the smaller the Rho value is, the smoother the curve is; the larger the Rho value, the fuller the curve is; when Rho < 0.5, the curve is ellipse; when Rho = 0.5, the curve is parabola; when Rho > 0.5, the curve is hyperbola. **(A)** The relationship between CXCL1 and the expression of immune invasion in BC. **(B)** The relationship between CXCL2 and the expression of immune invasion in BC.

The results suggested that both of them were negatively related to tumor purity. And they all had a negative correlation with macrophages and B cell. But they were positively correlated with T cell proliferator helper, T cell CD4^+^, Myeloid derived suppressor cells and Neutrophil. This indicated that they were closely related to cellular immunity. The difference between CXCL1-2 was that CXCL2 was positively correlated with cancer associated fibroblast and negatively correlated with Tregs, but CXCL1 had no significant relationship with them. What attracted our attention was that CXCL1-2 were associated with the increase of NK cell, but there was no significant relationship. This was consistent with the fact that NK cells in patients after chemotherapy were in a static state and participate in tumor immune function stably. The specific parameters information is shown in **Figure 6**.

### Neighbor Genes’ Network, Functional Enrichment Analysis of CXCL1 and CXCL2 in BC

**Figure 7:**
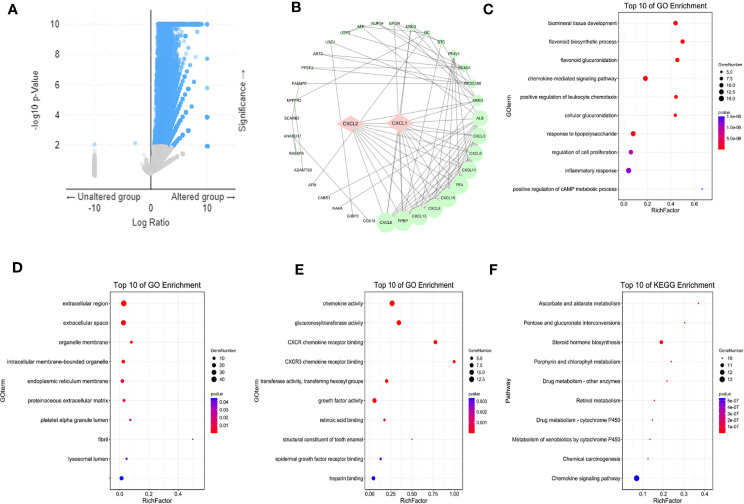
Markers and their adjacent genes regulatory networks. **(A)** Adjacent genes associated with markers from cBioPortal. **(B)** A PPI network from STRING. **(C–E)** The top ten GO BP, GO CC, GO MF from Gene Ontology. **(F)** The top ten KEGG pathways from KEGG. Isolate adjacent genes related to the differentially expressed CXC chemokines, GO and KEGG Pathway enrichment used students’ t-test, and *P* < 0.05 was the significance threshold.

In order to find out how the two biomarkers played a role in BC, cBioPortal was used to isolate adjacent genes related to the differentially expressed CXC chemokines (**Figure 7A**). It should be noted that this and our previous analysis of differentially expressed genes related genes and pathways had a mutual verification role. After removing the repetitive relationship between genes, a “protein-protein-interaction” (PPI) network was visualized by Cytoscape3.6.1 (**Figure 7B**). The corresponding genes were imported into David 6.8 software to annotate their functions and related pathways. **Figures 7C–E** showed the top 10 most abundant GO (BP, CC, MF) entries, respectively. Among the 10 highly abundant functions of BP class, the chemokine mediated signaling pathway, positive regulation of leukocyte chemotaxis, inflammatory response, regulation of cell promotion, were directly related to the occurrence and development of BC. Cellular glucuronidation and the positive regulation of camp metallic process were also indirectly related to BC. Extractive region, extractive space, organelle membrane, intracellular membrane bound organelle, endophytic reticulum membrane, proteinaceous extracellular matrix, platelet alpha granule lumen, fiber, lysosomal lumen were the top 10 entries in CC class. As expected, CXCL1-2 and their adjacent genes were mainly rich in chemokine activity, CXCR chemokine receptor binding and CXCR3 chemokine receptor binding in the molecular functional MF class. We expanded the adjacent genes to 200 and analyzed the KEGG pathway to expand the scope of the designed pathway. The results showed that among the top 10 KEGG signaling pathways, chemical carcinogenesis and chemokine signaling pathway were closely related to the occurrence and development of BC (**Figure 7F**), which were the same as the results of the top 50 targets. Interestingly, most of the other pathways involved glucose and drug metabolism suggested that CXCL1-2 might be related to the occurrence and development of BC, thus provided new ways for the treatment of BC. It was worth noting that these results had a lot of similarities with the previous correlation analysis of differentially expressed genes, which further showed the credibility of these data.

### Molecular Docking and Analysis

In order to find effective drugs of CXCL1-2, seven natural compounds which had been proved to have effect on BC *in vivo* and *in vitro* were screened *via* Drug Bank and TCMSP databases. Their structures are shown in **Supplementary Picture Figure 1**. Next, AutoDockTools-1.5.6 and PyMOL were used to dock compounds with CXCL1-2. Before that, “Auto-Grid algorithm” was used to build a cubic grid. Then, 20 different conformations of each gene and compound were obtained and analyzed. The effective compounds and conformations were screened by the dock binding free energy. The specific docking energy and bonds of compounds with them are shown in **Tables 1** and **2**. The 3D images and binding sites of CXCL1-2 are shown in **Supplementary Picture Figures 2** **–** **5** respectively.

**Table 1:** Dock binding free energies(^△^Gb) and bonds of the proteins.

Gene	Compounds	^△^Gb(kcal/mol)	Bonds formed between functional groups of components and proteins’ residues		
			Functional groups	Proteins’ residues	bond
	Strychnine	-7.71	O	GLU29	H-bond
	Quercetin	-6.94	O O H H	THR-37 CYS-9 CYS-9 HIS-33	H-bond H-bond H-bond H-bond
	Saikosaponin D	-6.74	O	PHE-98	H-bond
CXCL1	Puerarin	-5.85	O O	A: GLU-29 B: GLU-29	H-bond H-bond
	Baicalein	-5.39	H O O	SER-30 GLU-29 ILE-22	H-bond H-bond H-bond
	Syringin	-5.23	O O H	ILE-10 PHE-17 PHE-17	H-bond H-bond H-bond
	Curcumin	-4.72	O	TRP-47	H-bond

**Table 2:** Dock binding free energies(^△^Gb) and bonds of the proteins.

Gene	Compounds	^△^Gb(kcal/mol)	Bonds formed between functional groups of components and proteins’ residues		
			Functional groups	Proteins’ residues	bond
	Quercetin	-8.85	O H	LEU-45 ASP-1	H-bond H-bond
	Strychnine	-8.23	O O H	GLY-42 GLN-39 GLY-41	H-Bond H-bond H-bond
CXCL2	Baicalein	-8.16	H H O O H H	GLY-42 LEU-45 PHE-98 TRP-47 PRO-96 ASP-1	H-bond H-bond H-bond H-bond H-bond H-bond
	Puerarin	-7.54	O O H H	ASP-1 PRO-96 TRP-47 ASN-63	H-bond H-bond H-bond H-bond
	Syringin	-6.92	O O H O H O H	ASN-63 TRP-47 ASP-1 PRO-96 LEU-45 LEU-89 GLY-99	H-bond H-bond H-bond H-bond H-bond H-bond H-bond
	Saikosaponin D	-5.99	O and H O	PHE-98 MET-4	H-bond H-bond
	Curcumin	-4.72	O	TRP-47	H-bond

The results showed that the seven compounds had strong binding ability to both of them. It was not difficult to find that Quercetin and Strychnine had the lower docking energy with them than the others. In addition, GLU29 of CXCL1, ASP-1, PRO-96, TRP-47 and LEU-45 of CXCL2 could bind with several compounds. This provided 5 reliable amino acid sites for our later research. At the same time, a model with OB > 30% and DL > 0.18 was established to determine whether they had the potential to be developed as drugs, initially. As shown in **Table 3**, Quercetin was the most suitable compound for this model.

**Table 3:** Information about compounds.

Common name	Molecule ID	PubChem CID	OB (%)	DL
Saikosaponin D	MOL004637	107793	34.39	0.09
Puerarin	MOL012297	5281807	24.03	0.69
Syringin	MOL000347	5316860	14.64	0.32
Quercetin	MOL000098	5280343	46.43	0.28
Curcumin	MOL000090	5281767	5.15	0.41
Baicalein	MOL002714	5281605	33.52	0.21
Strychnine	MOL003429	441071	7.98	0.48

### ADMET Information of Quercetin

However, in order to get the exact result, its ADMET parameters were analyzed systematically. The absorption results showed that the water solubility and skin permeability of Quercetin were poor, but its absorption and utilization in the intestinal tract were good. As a substrate of PGP rather than an inhibitor of P-glycoprotein I/II, it could be transported by Pgp, which was the advantage of it. Its distribution results showed that it could not pass through the central nervous system (log Ps < -3) and was poorly distributed in the brain (log BB < +1). The results of VDS showed that the concentration of Quercetin in tissues and plasma was evenly distributed, thus avoiding the effects of renal failure and dehydration (-0.15 < VDS < 0.45). Most of the drugs in plasma were in an unbound state or a balance state with serum proteins. The degree of binding was closely related to whether they can effectively pass cells or spread. Unfortunately, the metabolic results showed that Quercetin was not the two majorly isomorphic 2D6 and 3A4 substrates of P450. But it was not a P450 inhibitor either. Therefore, its metabolic mode and related products needed to be further elucidated *in vivo* experiments. Although it was not the substrate of organic cation transporter 2 (OCT2), but its drug clearance was 0.407 (log ml/min/kg) by the ratio constant (CLTOT), which proved that it could be excreted through the hepatic space (metabolism of liver and biliary space) and renal space (excretion through kidney). This result was consistent with the bioavailability of Quercetin in our previous analysis. Finally, the toxicity test showed that Quercetin was not carcinogenic and had no toxicity to liver and skin. At the same time, the maximum tolerated dose (MRTD) of it was 0.499 (log mg/kg/day), which provided a valuable reference for future clinical research on it.

Based on the above analysis, Quercetin conformed to the ADMET principle and had the possibility of being developed into a drug. All the information is shown in **Table 4**.

**Table 4:** Information about Quercetin.

Property	Model Name	Predicted Value
Absorption	Water solubility	-2.925
	Caco2 permeability	-0.229
	Intestinal absorption (human)	77.207
Distribution	Skin Permeability	-2.735
	P-glycoprotein substrate	Yes
	P-glycoprotein I inhibitor	No
	P-glycoprotein II inhibitor	No
	VDss (human)	1.559
	Fraction unbound (human)	0.206
Metabolism	BBB permeability	-1.098
	CNS permeability	-3.065
	CYP2D6 substrate	No
	CYP3A4 substrate	No
	CYP1A2 inhibitor	Yes
	CYP2C19 inhibitor	No
	CYP2C9 inhibitor	No
Excretion	CYP2D6 inhibitor	No
	CYP3A4 inhibitor	No
	Total Clearance	0.407
	Renal OCT2 substrate	No
Toxicity	AMES toxicity	No
	Max. tolerated dose (human)	0.499
	hERG I inhibitor	No
	hERG II inhibitor	No
	Oral Rat Acute Toxicity (LD50)	2.471
	Oral Rat Chronic Toxicity (LOAEL)	2.612
	Hepatotoxicity	No
	Skin Sensitisation	No
	T. Pyriformis toxicity	0.288
	Minnow toxicity	3.721

### Quercetin Decreases Proliferation of MDA-MB-231 and MCF-7 Cells

Before verifying the relationship between Quercetin and CXCL1-2, it is necessary to verify its effect on BC. In order to avoid drug specificity, MDA-MB-231 and MCF-7 cells were included in the study.

**Figure 8:**
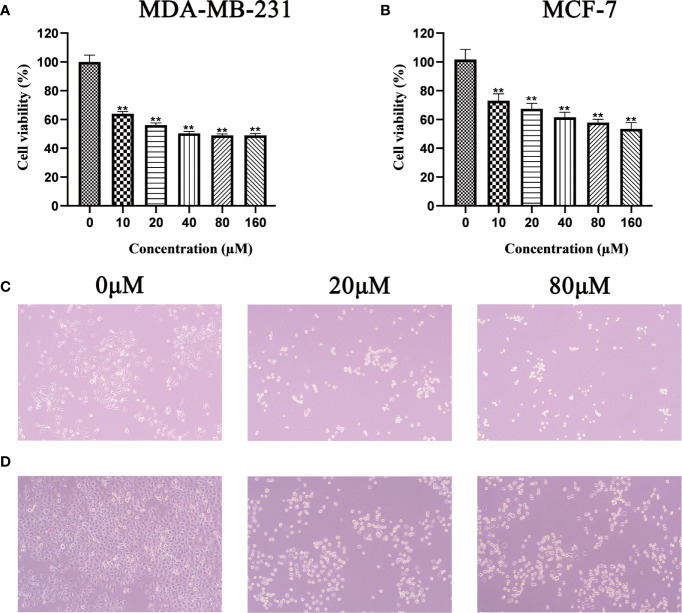
Effect of Quercetin on the viability of MDA-MB-231 **(A)** and MCF-7 **(B)** cells, after treatment with Quercetin for 24 h, the viability of these cells was measured by MTT assay, **p < 0.01 vs. control. Under 10x microscope, the cell morphology of different groups in the MDA-MB-231 **(C)** and MCF-7 **(D)** cells was observed.

MTT results showed that as the concentrations of Quercetin increased, cell viability was significantly decreased in MDA-MB-231 and MCF-7 cells compared with that in the control (*p*<0.05 and *p*<0.01). The half-maximal inhibitory concentration (IC50) of Quercetin for MCF-7 cells was about 160μM and for MDA-MB-231 cells was about 100μM (**Figures 8A, B** **)**. At the same time, the cell morphology in the treatment group and the control group were photographed under 10x microscope. The results showed that different concentrations of Quercetin changed cell morphology and caused cell death (**Figures 8C, D** **)**.

### Quercetin Suppressed the Growth of MDA-MB-231 and MCF-7 Cells *In Vitro*

**Figure 9:**
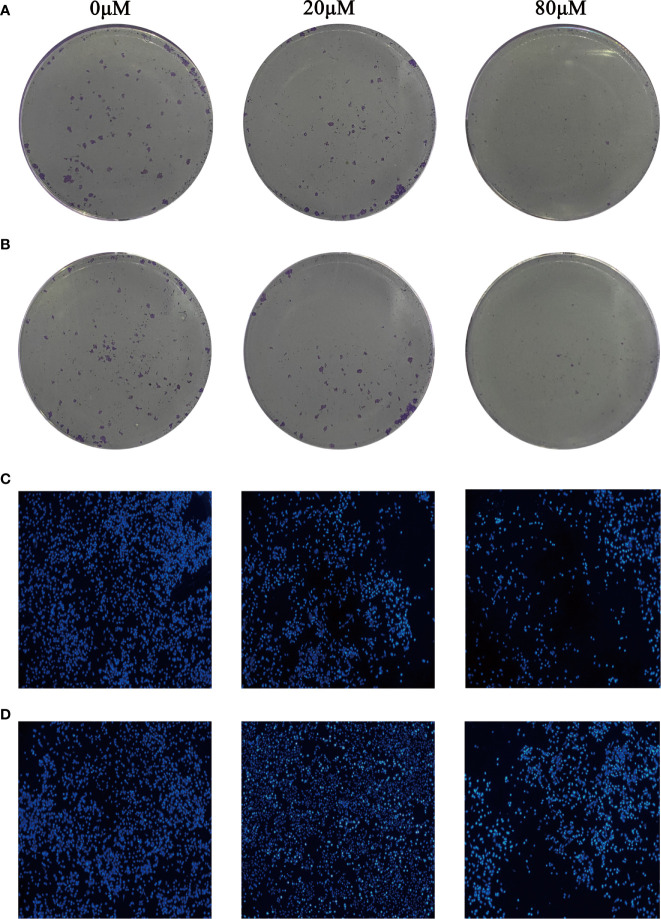
Quercetin suppressed the growth of MDA-MB-231 cells **(A)** and MCF-7 cells **(B)** in a dose-dependent manner. Quercetin increased MDA-MB-231 cells **(C)** and MCF-7 cells **(D)** apoptosis in a dose-dependent manner.

The results of plate cloning experiment showed that the number of MDA-MB-231 and MCF-7 cells clones gradually decreased with the increase of Quercetin concentration (**Figures 9A, B** **)**. So, Quercetin suppressed the growth of MDA-MB-231 and MCF-7 cells *in vitro*.

### Quercetin Increased MDA-MB-231 and MCF-7 Cells Apoptosis *In Vitro*

Hoechst 33342 staining was used to identify whether Quercetin increased MDA-MB-231 and MCF-7 cells apoptosis. The results showed that compared with control group, apoptosis in MCF-7 and MDA-MB-231 cells treated with different concentrations of Quercetin were markedly increased (**Figures 9C, D** **)**.

### Quercetin Increased the Expression of CXCL1 and CXCL2 in BC

**Figure 10:**
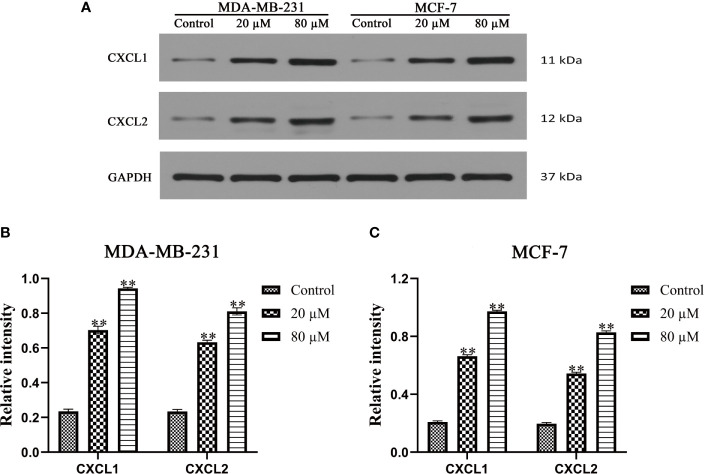
The expression of CXCL1 and CXCL2 was determined by Western blot. **(A)** The expression of CXCL1 and CXCL2 was determined by Western blot. **(B)** Quercetin increased the expression of CXCL1-2 in MDA-MB-231 cells in a dose-dependent manner. **(C)** Quercetin increased the expression of CXCL1-2 in MCF-7cells in a dose-dependent manner. **p < 0.01 vs. the Control group.

**Figure 11:**
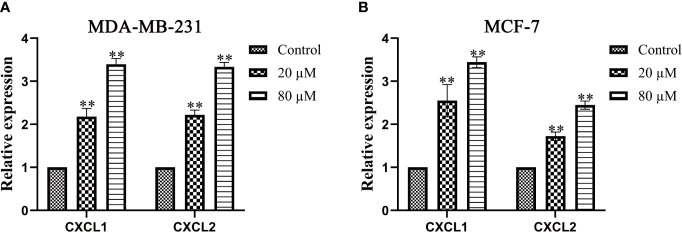
The expression of CXCL1 and CXCL2 was determined by Real-Time PCR. **(A)** Quercetin increased the expression of CXCL1-2 in MDA-MB-231 cells in a dose-dependent manner. **(B)** Quercetin increased the expression of CXCL1-2 in MCF-7cells in a dose-dependent manner. **p < 0.01 vs. the Control group.

Western blot and Real-Time PCR were used to verify the effect of Quercetin on the expression of CXCL1-2. As shown in **Figures 10A**–**C**, a significantly increased expression of CXCL1-2 in MDA-MB-231 and MCF-7 cells was observed in Quercetin groups when compared with the control group (*p*<0.05 or *p*<0.01). And Real-Time PCR results showed that a significantly increased expression of CXCL1-2 in MDA-MB-231 and MCF-7 cells was observed in Quercetin groups when compared with the control group (*p*<0.05 or *p*<0.01), as shown in **Figures 11A, B**. The information of related primers is shown in **Supplementary Table 1**.

## Discussion

Breast cancer, as a kind of malignant tumor disease threatening the life and health of women all over the world, needs accurate biomarkers to judge it urgently. CXC chemokines, as very important inflammatory mediators, are closely related to the maturation, differentiation and transportation of leukocytes. More and more evidences show that the expression patterns of chemokines change from different cell types, which play an important role in the proliferation, apoptosis and metastasis of tumor cells (11). The expression levels of CXC chemokines in many kinds of tumors have been studied, but their prognostic value and biological function in BC have not been well described (34). At the same time, the correlation between CXC chemokines and tumor microenvironment also varies with the type of tumor. Therefore, it is of great significance to elucidate the relationship between CXC chemokines and immune cells in BC.

In this study, the expression of CXC chemokines in BC were first investigated. Results showed that eight genes were differentially expressed in it. Among them, CXCL1-3 and CXCL12 were down-regulated, while CXCL9-11 and CXCL13 were up-regulated. However, these results were different from the expression levels of CXC chemokines in many other tumors, which suggested that the expression and mechanisms of CXC chemokines in tumors were diverse. In addition, the relationship between differentially expressed genes and tumor staging were also studied. The results showed that the expression of CXCL1-2 and CXCL12 decreased with the development of tumor, while CXCL13 was opposite. The mutation of gene played a very important role in the occurrence and development of tumors. The mutation results of CXCL1-2 in the database confirmed it. The correlation between the differentially expressed genes and the survival rate of BC patients was also worthy of our study. We found that the overall survival rate of BC patients with low expression of CXCL1-2 and CXCL13 were lower, consistent with the results of gene mutation. These data suggested that CXCL1-2 could be used as new biomarkers of BC.

In order to understand the role of CXCL1-2 in BC, GO enrichment analysis and KEGG pathway enrichment analysis were used to study their functions. What’s more, we found that the functions of these genes were not only related to chemical carcinogenesis and chemokine signaling pathway, but also related to glucose and drug metabolism. The glycolysis pathway involved in glucose metabolism has long been confirmed to be involved in the development of tumor, and drug autogenous metabolism plays an important and decisive role in the treatment of diseases. The potential relationship between CXCL1-2 and these results provided a new direction for our later research.

Immune infiltration is closely related to the occurrence and development of tumor (35). Chemotherapy and drug therapy can change the immune capacity of patients, thus further affecting the survival and prognosis of patients. Therefore, immunotherapy has become the first choice for the treatment of a variety of cancers (36). This study analyzed the correlation between the expression of CXCL1-2 and tumor purity, T cell CD8^+^, T cell CD4^+^, Macrophage infiltration and other immune cells. The results showed that both of them were negatively correlated with tumor purity, and were significantly correlated with T cell promoter helper, T cell CD4^+^, Myoid derived suppressor cells, Neutrophil, Macrophages and B cell. To our surprise, both CXCL1-2 increased CD4^+^/CD8^+^, but negatively correlated with Macrophages and B cells. This suggested that they reflected the immune status mainly through the action of T cells and Neutrophil, and the relationship between them and T cell proliferator helper also illustrated this point. CXCL1-2 were produced by immune cells such as Neutrophils, which was consistent with our conclusion. Therefore, they must have an inseparable relationship with tumor immunity, which has been confirmed in a variety of tumors (37–39). At present, tumor associated neutrophils (TANs) are considered to have two sides of promoting tumor and anti-tumor, and most of the research results are biased towards the tumor promoting effect of TANs. However, we think that the anti-tumor effect of TANs should not be ignored. Recently, Fridlander found that compared with the mice without SM 16, after blocking the TGF-β signaling pathway with SM 16, the administration group increased the number of TANs in the tumor, slowed down the tumor growth, induced the activation of anti-tumor effect of CD8+ T cells, and induced the aggregation of Neutrophils (40). After being activated, these Neutrophils killed tumor cells *in vitro*, showing an anti-tumor “N1” type. And it promoted the proliferation of tumor cells when the anti-LY6G monoclonal antibody was used to eliminate these cytotoxic neutrophils (41).

At the same time, analysis of the above results showed that CXCL1-2 were significantly positively correlated with tumor associated fibroblasts, and we thought that they might have a greater correlation with BC. Therefore, we intended to screen compounds for direct interaction with them through molecular docking and ADMET. Results showed that Quercetin (42), Strychnine (43), Baicalein (44), Puerarin (45), Syringin (46), Saikosaponin D (47) and Curcumin (48) all had good binding ability with CXCL1-2. Based on the comprehensive analysis of the binding energies of the compounds with CXCL1-2 and their OB and DL values, we found that Quercetin most likely to be developed as drugs that interacted directly with CXCL1-2. And our study also provided the binding sites of each compound with CXCL1-2. It was obvious that GLU29 of CXCL1, ASP-1, PRO-96, TRP-47 and LEU-45 of CXCL2 were the most potential sites, which provided valuable reference for further study of pharmacodynamics and mechanism. In addition, we confirmed the inhibitory effect of Quercetin on proliferation and promoting apoptosis of BC related cell lines *in vitro*. Western blot and Real-Time PCR confirmed that it increased the expression of CXCL1-2 in protein and mRNA.

Many studies have found that N1 type TANs induce tumor cell apoptosis (49), which is also consistent with our conclusion. In addition, Neutrophils secrete hydrogen peroxide after direct contact with cancer cells, leading to tumor cell death through Ca2^+^ influx in TRPM2–CA2^+^ channel (50). Sun also found that Neutrophils isolated from healthy people promoted tumor cell apoptosis and inhibited tumor growth through Fas ligand/Fas pathway (51, 52). However, the role of CXCL1-2 has not been reported. Although the dual nature of Neutrophils makes them difficult to explain their role in tumor. However, we believe that in breast cancer, CXCL1-2 secreted and recruited by Neutrophils will initially come to the tumor cell area with them, so as to achieve the efficacy of killing tumor cells. However, with the change of tumor cycle, they are also killed or utilized by tumor cells, thus losing their original functions. As we discussed earlier, with the change of tumor cycle, their expression constantly decreased, so they couldn’t cooperate with the corresponding immune cells, and couldn’t recognize and kill tumor cells. But the specific conclusion should be based on the test results of more clinical samples, not based on this article. Therefore, we are now confirming the above conclusion based on more clinical samples. On the basis of confirmation, our research on Quercetin in breast cancer, especially the relationship between quercetin and CXCL1-2, will be carried out in detail.

## Conclusion

Taken together, our results showed that two down-regulated genes CXCL1-2 were related with poor prognosis and immune infiltration in BC. Therefore, CXCL1-2 might be new biomarkers and potential therapeutic targets for patients with BC. In addition, Quercetin was likely to be a targeted drug for CXCL1-2. Experiments *in vitro* also confirmed the inhibitory effect of Quercetin on proliferation and promoting apoptosis of the two cell lines. Western blot and Real-Time PCR also confirmed that Quercetin increased the expression of CXCL1-2. However, our research has some limitations. More studies *in vitro* and *in vivo* are needed to verify our results.

## Data Availability Statement

The datasets presented in this study can be found in online repositories. The names of the repository/repositories and accession number(s) can be found below: https://pubchem.ncbi.nlm.nih.gov/, http://www.oncomine.org, http://gepia.cancer-pku.cn/index.html, http://ualcan.path.uab.edu/analysis.html, http://www.cbiobortal.org, https://cistrome.shinyapps.io/timer/.

## Author Contributions

FW and CY designed the study. FW, CY, Y-FY, H-ZW and BL performed the research. Y-FY, H-ZW and BL collected and analyzed the data. FW and CY wrote the paper. All authors contributed to the article and approved the submitted version.

## Funding

This work was supported by National key R & D plan key project of TCM Modernization Research (2017YFC1701000).

## Conflict of Interest

The authors declare that the research was conducted in the absence of any commercial or financial relationships that could be construed as a potential conflict of interest.

## Supplementary Material

Supplementary Picture
**Figure 1** 2D structures of seven compounds.

Supplementary Picture
**Figure 2** 3D diagrams of the docking results between compounds and CXCL1. **(A)** Saikosaponin D. **(B)** Purerarin. **(C)** Syringin. **(D)** Quercetin.

Supplementary Picture
**Figure 3** 3D diagrams of the docking results between compounds and CXCL1. **(A)** Curcumin. **(B)** Baicalein. **(C)** Strychnine.

Supplementary Picture
**Figure 4** 3D diagrams of the docking results between compounds and CXCL2. **(A)** Saikosaponin D. **(B)** Purerarin. **(C)** Syringin. **(D)** Quercetin.

Supplementary Picture
**Figure 5** 3D diagrams of the docking results between compounds and CXCL2. **(A)** Curcumin. **(B)** Baicalein. **(C)** Strychnine.
