# Supplementary material for: Bioinformatics, Molecular Docking and Experiments In Vitro Analyze the Prognostic Value of CXC Chemokines in Breast Cancer
Source: Front Oncol. 2021 May 26;11:665080. doi: 10.3389/fonc.2021.665080 (PMC8189319; doi:10.3389/fonc.2021.665080)
Supplement: Supplementary Picture — Figure 1 2D structures of seven compounds. [file DataSheet_1.docx]

Supplementary Table and Figures

**Supplementary Table 1.** Information about primers.

| Primers | Base sequence (5`-3`) | | Tm | CG% | Length |
| --- | --- | --- | --- | --- | --- |
| H-GAPDH | sense | CATCATCCCTGCCTCTACTGG | 59.4 | 57.1 | 259 |
|  | antisense | GTGGGTGTCGCTGTTGAAGTC | 60.1 | 57.1 |  |
| H-CXCL1 | sense | AACCGAAGTCATAGCCACACTC | 58.5 | 50.0 | 251 |
|  | antisense | CTTCTCCTAAGCGATGCTCAAA | 59.4 | 45.5 |  |
| H-CXCL2 | sense | AAGGGGTTCGCCGTTCTC | 59.6 | 61.1 | 232 |
|  | antisense | TGGCAGCGCAGTTCAGTG | 59.3 | 61.1 |  |


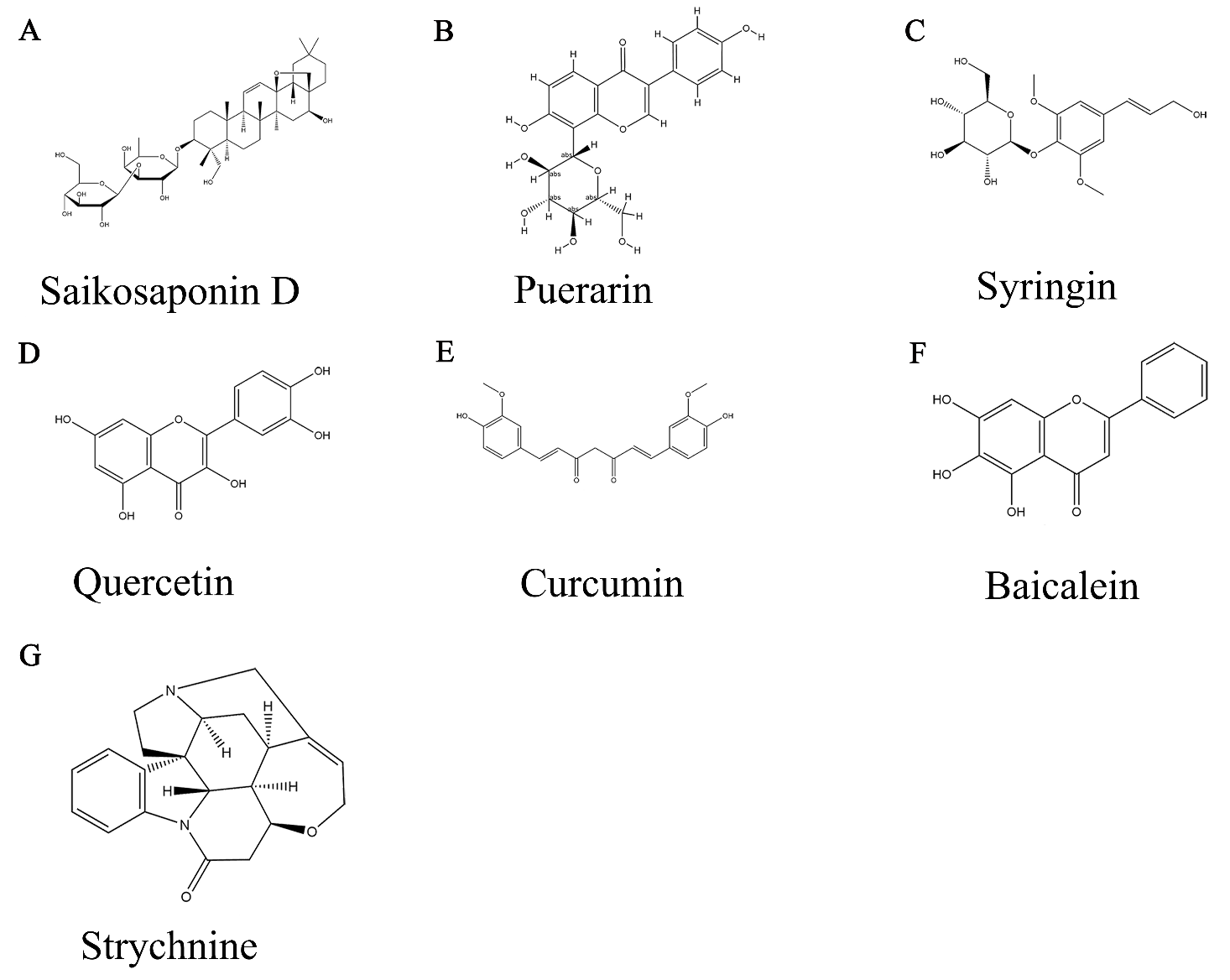


**Supplementary Figure 1.** 2D structures of seven compounds.


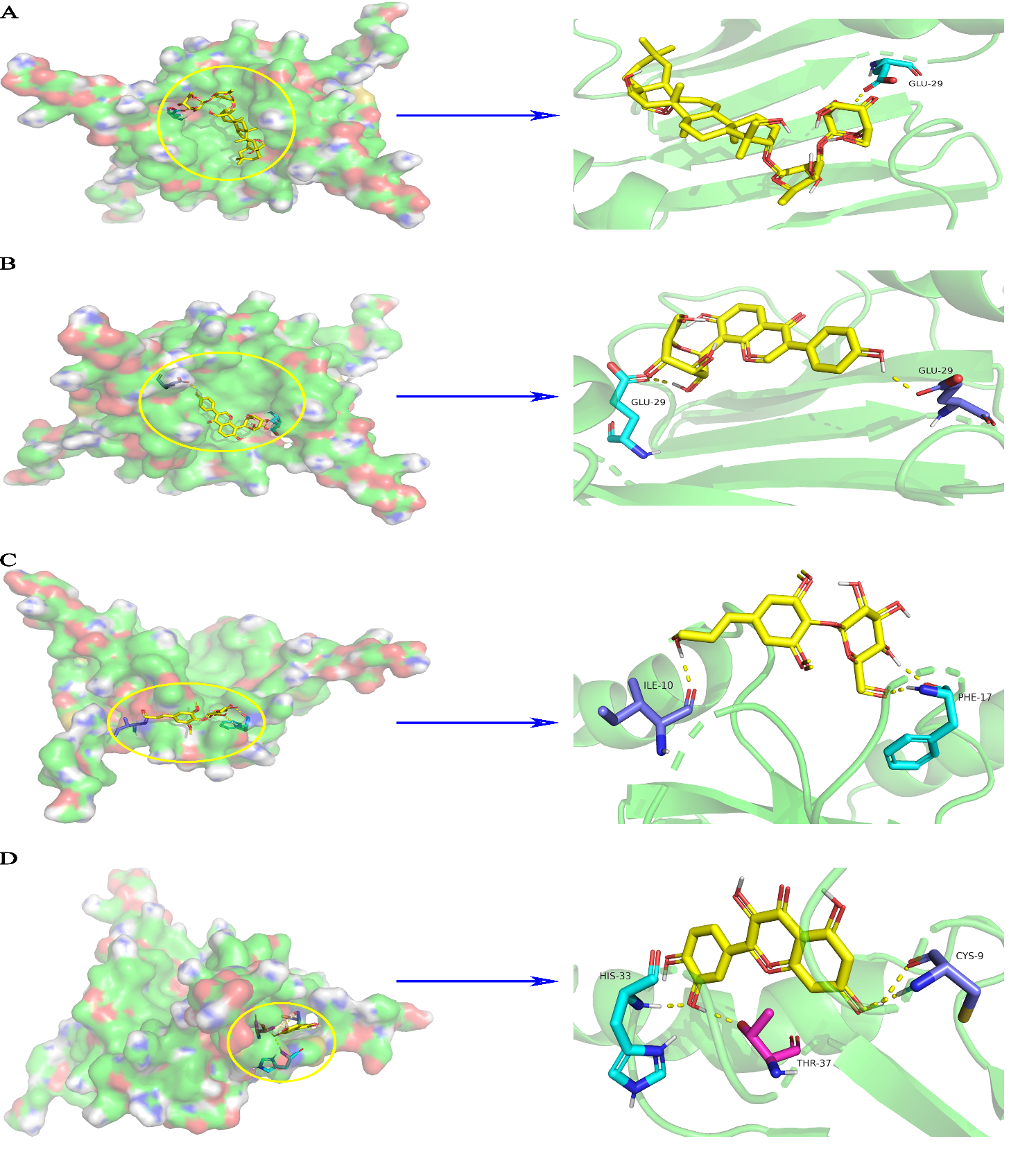


**Supplementary Figure 2.** 3D diagrams of the docking results between compounds and CXCL1.

**(A)** Saikosaponin D. **(B)** Purerarin. **(C)** Syringin. **(D)** Quercetin.


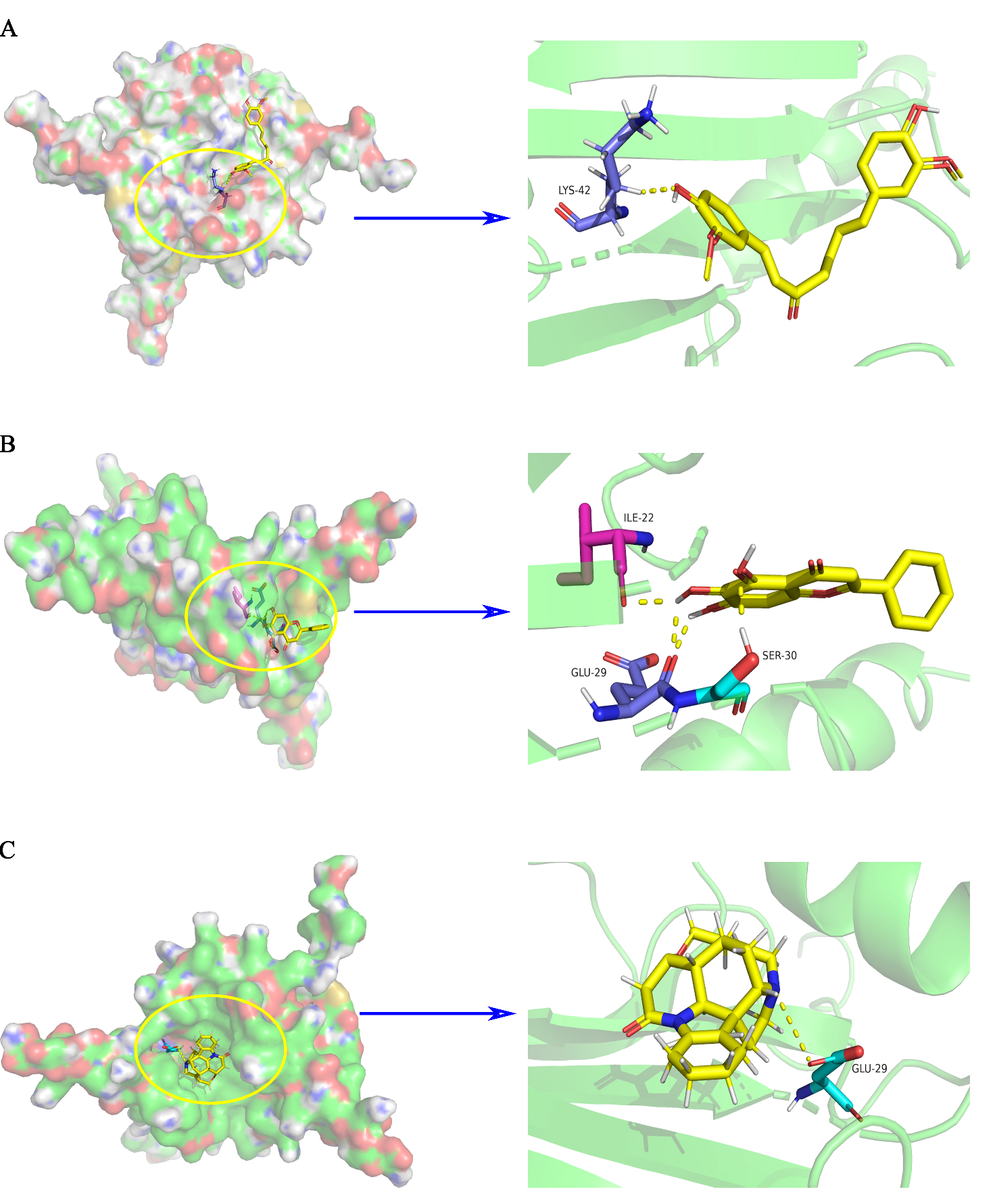


**Supplementary Figure 3.** 3D diagrams of the docking results between compounds and CXCL1. **(A)** Curcumin. **(B)** Baicalein. **(C)** Strychnine.


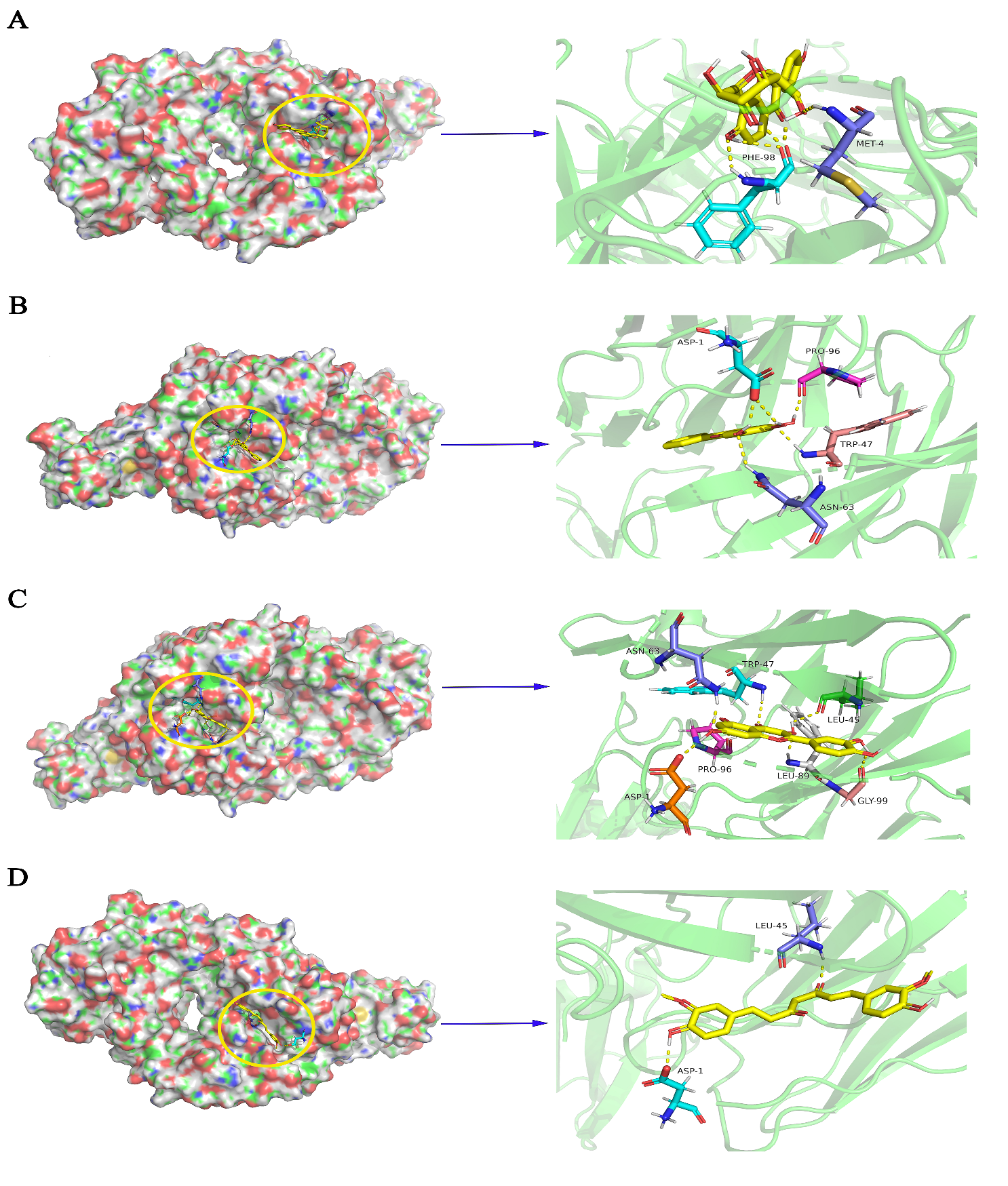


**Supplementary Figure 4.** 3D diagrams of the docking results between compounds and CXCL2.

**(A)** Saikosaponin D. **(B)** Purerarin. **(C)** Syringin. **(D)** Quercetin.


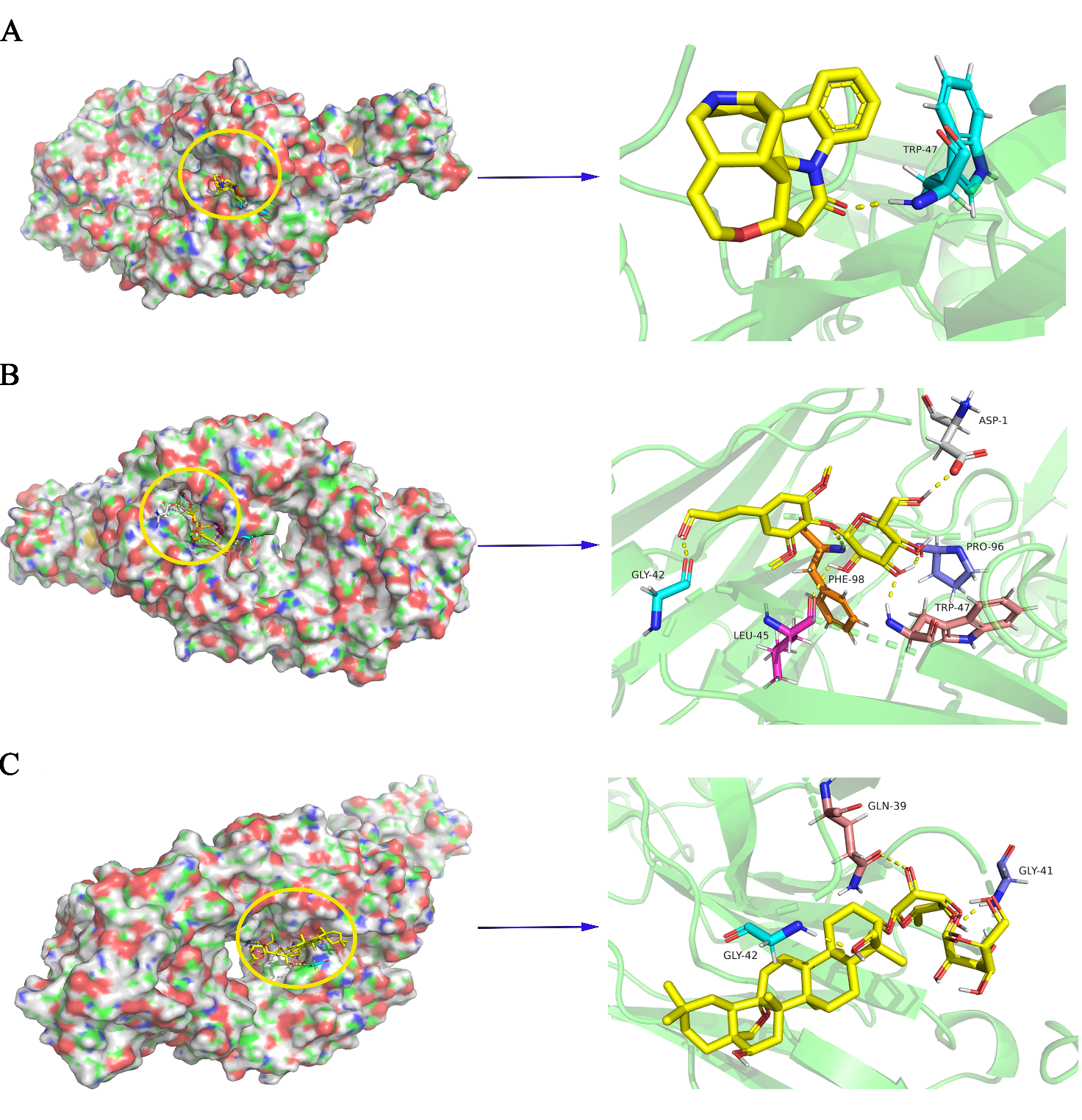


**Supplementary Figure 5.** 3D diagrams of the docking results between compounds and CXCL2. **(A)** Curcumin. **(B)** Baicalein. **(C)** Strychnine.
